# Supplementary material for: Content-rich biological network constructed by mining PubMed abstracts
Source: BMC Bioinformatics. 2004 Oct 8;5:147. doi: 10.1186/1471-2105-5-147 (PMC528731; doi:10.1186/1471-2105-5-147)
Supplement: Additional File 2 — The original results of the above study (non-essential files are deleted to keep the file size under the limit set by BMC bioinformatics). [file 1471-2105-5-147-S2.bz2 › chilibotAdditionalFile2/dip30/27ID8999548E90/html/CASP1_IL18.html]

 


 **CASP1** and **IL18** 
  
Found 147 abstracts in PubMed, retrieved 30.  
 

 What does Google say? 
 PDF only 
| .edu only 

---

**Interactive relationship** (e.g. stimulation, inhibition, etc)

**Neutral relationship**- Our results show that infection by S. typhimurium causes caspase 1  [ **CASP1** ]  dependent activation of IL 18  [ **IL18** ]  and triggers the release of IL 18  [ **IL18** ]  in human DCs.  Ref: 12377944 J Leukoc Biol, 2002
- Recent publications have demonstrated that the protease caspase 1  [ **CASP1** ]  is responsible for the processing of pro interleukin 18  [ **IL18** ]  IL 18  [ **IL18** ]  into the active form.  Ref: 12874316 Infect ImmunInfect Immun, 2002
- In addition, splenocytes from IL 10 mice produced elevated levels of nitric oxide NO compared to WT mice, and NO has been shown to inhibit activity of interleukin 1 converting enzyme ICE  [ **CASP1** ] , which is required for IL 18  [ **IL18** ]  production.  Ref: 12670447 Cytokine, 2003
- Interleukin 18  [ **IL18** ]  IL 18  [ **IL18** ]  is a proinflammatory cytokine expressed in microglia and processed by caspase 1  [ **CASP1** ] .  Ref: 12122053 J Neurosci, 2002
- When the cells were lysed in the presence of dithiothreitol, caspase 1  [ **CASP1** ]  precursor became active and converted pro IL 18  [ **IL18** ]  into mature IL 18  [ **IL18** ] .  Ref: 12706898 Gene, 2003
- Caspase 1  [ **CASP1** ]  is required for cleavage of the inactive precursor form of IL 18  [ **IL18** ]  into an active cytokine, and caspase 1  [ **CASP1** ]  deficient mice are resistant to lethal endotoxemia.  Ref: 12792854 J Infect Dis, 2003
- The release of IL 18  [ **IL18** ]  from mammalian cells is linked to its proteolytic activation by caspases including interleukin 1 converting enzyme ICE  [ **CASP1** ] .  Ref: 12597888 Protein Expr Purif, 2003
- As previously reported, transgenic mice KCASP1Tg, that over express caspase 1  [ **CASP1** ]  in their KC, display high serum levels of IgE, and spontaneously develop chronic dermatitis by production of IL 18  [ **IL18** ]  and IL 1beta.  Ref: 12697661 Int Immunol, 2003
- In addition, we confirmed that it is functionally active form of IL 18  [ **IL18** ]  using the inhibitor of caspase 1  [ **CASP1** ] .  Ref: 12387830 Biochem Biophys Res Commun, 2002
- We demonstrate that activation and release of IL 18  [ **IL18** ]  are blocked by mutations in the Salmonella sipB gene, which encodes a virulence factor that activates caspase 1  [ **CASP1** ]  to induce apoptosis.  Ref: 12377944 J Leukoc Biol, 2002
- HBcAg dependent IL 18  [ **IL18** ]  levels were abrogated by inhibition of Caspase 1  [ **CASP1** ] , but not by blockade of CD40 CD154 interaction.  Ref: 12858406 J Med Virol, 2003
- Mice lacking caspase 1  [ **CASP1** ]  exhibited both improved peri infarct survival and a decreased rate of ventricular dilatation, possibly due in part to a decrease in MMP 3 activity, IL 18  [ **IL18** ]  production, and a reduction in the rate of apoptosis after experimental MI.  Ref: 12788386 J Mol Cell Cardiol, 2003

**Non-interactive relationship** (e.g. studied together, co-existance, homology, etc.)

- Compared with untreated OA cartilage, oral treatment with L NIL significantly decreased ICE  [ **CASP1** ]  and IL 18  [ **IL18** ]  levels in cartilage from the femoral condyles and tibial plateaus, to values similar to those in normal dogs.  Ref: 12384922 Arthritis Rheum, 2002
- Surprisingly, this mRNA expression did not translate into intracellular accumulation of IL 1beta or IL 18  [ **IL18** ]  precursor proteins or secretion of mature cytokines, despite the presence of detectable caspase 1  [ **CASP1** ]  activity in these cells.  Ref: 12433285 J Interferon Cytokine Res, 2002
- To investigate the intra hepatic activation of the IFN system in patients affected by chronic HCV infection in comparison with that observed in a non infectious liver disease such as non alcoholic steatohepatitis, we measured the liver steady state mRNA levels of interferon alpha, interferon beta and interferon gamma as well as of IFN related genes IFNAR 1, STAT1alpha, PKR, 2 5 AS, IRF 1, ICE  [ **CASP1** ]  and IL 18  [ **IL18** ] .  Ref: 12794720 J Med Virol< MedlineTA>J Med Virol, 2002
- The expression of mRNA protein for caspase 1  [ **CASP1** ]  and IL 18  [ **IL18** ]  in brain homogenates increased progressively at 12 hr to 14 d after HI.  Ref: 12122053 J Neurosci, 2002
- COS 7 cells transfected with plasmids encoding pro IL 18  [ **IL18** ]  and caspase 1  [ **CASP1** ]  precursor expressed the two proteins intracellularly.  Ref: 12706898 Gene, 2003
- Although IL 12 is also an IFN gamma inducing factor  [ **IL18** ] , the activity of IL 18  [ **IL18** ]  but not IL 12 in models of sepsis and death is dependent on the intracellular cysteine protease IL 1beta converting enzyme caspase 1  [ **CASP1** ] .  Ref: 12792854 J Infect Dis, 2003
- After MI, enhanced cardiac activity of the pro IL 18  [ **IL18** ]  processing enzyme, caspase 1  [ **CASP1** ] , was measured.  Ref: 12829183 Cardiovasc Res, 2003
- Our aim was to characterize the expression of IL 18  [ **IL18** ]  and its receptor in relation to caspase 1  [ **CASP1** ]  and IL 1beta after HI and to evaluate to what extent IL 18  [ **IL18** ]  contributes to HI brain injury.  Ref: 12122053 J Neurosci, 2002
- Remarkably, caspase 1  [ **CASP1** ]  activity and IL 18  [ **IL18** ]  were significantly increased in the kidney in neutrophil depleted mice with ARF.  Ref: 12393844 J Clin Invest, 2002
- Notably, stenotic lesions, but not AAAs, contained mature forms of the interferon gamma inducing cytokines IL 12 and IL 18  [ **IL18** ]  as well as the IL 18  [ **IL18** ]  processing enzyme caspase 1  [ **CASP1** ] .  Ref: 12163375 Am J Pathol, 2002
- Interleukin 18  [ **IL18** ]  IL 18  [ **IL18** ]  was also studied and served as reference for the effects of ICE  [ **CASP1** ] .  Ref: 12384922 Arthritis Rheum, 2002
- Taken together, these results indicate that HBcAg induces IL 18  [ **IL18** ]  secretion by induction of Caspase 1  [ **CASP1** ] .  Ref: 12858406 J Med Virol, 2003
- The conclusion that G CSF inhibits caspase 1  [ **CASP1** ]  activity was supported bythe finding that the release of IL 18  [ **IL18** ]  was also inhibited by G CSF, similar to IL 1beta release.  Ref: 12115655 Eur J Immunol, 2002
- CONCLUSION This study demonstrated that in situ in OA cartilage, the stimulation of chondrocytes by NO is at least partly responsible for the up regulation of ICE  [ **CASP1** ]  and IL 18  [ **IL18** ]  synthesis while decreasing the level of the ICE  [ **CASP1** ]  inhibitor PI 9.  Ref: 12384922 Arthritis Rheum, 2002
- Because IL 18  [ **IL18** ]  directly stimulates T cells and mast cells to release AD associated molecules, Th2 cytokines, and histamine, we investigated the capacity of IL 18  [ **IL18** ]  to induce AD like inflammatory skin disease by analyzing KIL 18Tg and KCASP1Tg, which skin specifically overexpress IL 18  [ **IL18** ]  and caspase 1  [ **CASP1** ] , respectively.  Ref: 12151598 Proc Natl Acad Sci U S A, 2002
- To produce an authentic IL 18  [ **IL18** ] , we have devised a bicistronic expression system for the coupled transcription and translation of ProIL 18 with caspase 1  [ **CASP1** ]  ICE  [ **CASP1** ]  or caspase 4 ICE  [ **CASP1** ]  rel II, TX, ICH 2.  Ref: 12597888 Protein Expr Purif, 2003
- Two recombinant hybrid baculoviruses containing cDNA of the porcine precursor IL 18  [ **IL18** ]  and the porcine caspase 1  [ **CASP1** ]  were constructed and were used to infect silkworm larvae.  Ref: 12655117 J Vet Med Sci, 2003
- While statins may stimulate the secretion of caspase 1  [ **CASP1** ] , IL 1beta and IL 18  [ **IL18** ]  in peripheral mononuclear cells in response to Mycobacterium tuberculosis, they exhibit additional effects on inflammation by decreasing IL 6 synthesis in human vascular smooth muscle cells VSMC in vitro.  Ref: 12698208 Drugs Today Barc , 2003
- Mice with targeted deletion of caspase 1  [ **CASP1** ]  interleukin 1beta IL 1beta converting enzyme lack the active forms of IL 1beta and IL 18  [ **IL18** ] , two cytokines implicated in maladaptive ventricular remodeling following cardiac injury.  Ref: 12788386 J Mol Cell Cardiol, 2003
- Although IL beta plasma levels were not detectable, both IL 18  [ **IL18** ]  levels and the rate of apoptosis in remodeling, non infarcted muscle were significantly higher in WT compared to caspase 1  [ **CASP1** ]  KO animals.Conclusion.  Ref: 12788386 J Mol Cell Cardiol, 2003
- Caspase 1  [ **CASP1** ] , the prototypic caspase, is known to process the cytokines IL 1beta and IL 18  [ **IL18** ]  to mature forms but it is unclear whether, like other caspases, it can induce apoptosis by activation of downstream protease cascades.  Ref: 12444148 J Immunol, 2002
- At 12 weeks after surgery, cartilage from the femoral condyles and tibial plateaus were processed for immunohistochemistry for ICE  [ **CASP1** ] , IL 18  [ **IL18** ] , and protease inhibitor 9 PI 9, a natural inhibitor of ICE  [ **CASP1** ] , followed by morphometric analysis.  Ref: 12384922 Arthritis Rheum, 2002
- OPH 001 significantly reduced the increase in caspase 1  [ **CASP1** ]  activity and IL 18  [ **IL18** ]  and prevented neutrophil infiltration in the kidney during ischemic ARF.  Ref: 12393844 J Clin Invest, 2002
- RESULTS Both ICE  [ **CASP1** ]  and IL 18  [ **IL18** ]  were present in situ in the canine cartilage, with a significant increase in the level of these 2 proteins in OA cartilage.  Ref: 12384922 Arthritis Rheum, 2002
- Neutrophil independent mechanisms of caspase 1  [ **CASP1** ]  and IL 18  [ **IL18** ]  mediated ischemic acute tubular necrosis in mice.  Ref: 12393844 J Clin Invest, 2002
- Here, we report that hypoxic exposure of cultured brain microglia BV 2 mouse microglia cells and rat primary microglial cultures induces expression and activation of caspase 11, which is accompanied by activation of caspase 1  [ **CASP1** ]  and secretion of mature IL 1beta and IL 18  [ **IL18** ] .  Ref: 12829320 Brain Res Mol Brain Res, 2003
- The specific caspase 1  [ **CASP1** ]  inhibitor Ac YVAD CMK blocked the early IL 18  [ **IL18** ]  release in AM infected with the virulent strain.  Ref: 12874316 Infect ImmunInfect Immun, 2003
- IL 18  [ **IL18** ]  levels and the outcome of innate immune response to lipopolysaccharide importance of a positive feedback loop with caspase 1  [ **CASP1** ]  in IL 18  [ **IL18** ]  expression.  Ref: 12193723 J Immunol, 2002
- Gerbil interleukin 18  [ **IL18** ]  and caspase 1  [ **CASP1** ]  cloning, expression and characterization.  Ref: 12706898 Gene, 2003
- Having recently described the injurious role of caspase 1  [ **CASP1** ]  mediated production of the proinflammatory cytokine IL 18  [ **IL18** ]  in ischemic acute renal failure ARF, we report here on the effect of the newly developed caspase inhibitor Quinoline Val Asp Ome CH 2 OPH OPH 001 on caspase 1  [ **CASP1** ] , IL 18  [ **IL18** ] , neutrophil infiltration, and renal function in ischemic ARF.  Ref: 12393844 J Clin Invest, 2002
- Increased expression of caspase 1  [ **CASP1** ]  mRNA in response to the higher IL 18  [ **IL18** ]  levels induced at the 150 and 500 micro g, but not at the 75 micro g doses of LPS may represent a positive feedback regulatory loop leading to sustained serum IL 18  [ **IL18** ]  levels.  Ref: 12193723 J Immunol, 2002
- A widespread expression of caspase 1  [ **CASP1** ]  and IL 18  [ **IL18** ]  protein in microglia was found in the HI hemisphere.  Ref: 12122053 J Neurosci, 2002
- The mRNA for IL 18  [ **IL18** ]  and caspase 1  [ **CASP1** ]  were analyzed with reverse transcriptase PCR, protein was analyzed by Western blot IL 18  [ **IL18** ] , caspase 1  [ **CASP1** ]  or ELISA IL 1beta, and the regional distribution was assessed by immunohistochemistry.  Ref: 12122053 J Neurosci, 2002
- We are reporting the molecular cloning of gerbil interleukin 18  [ **IL18** ]  IL 18  [ **IL18** ]  and caspase 1  [ **CASP1** ] .  Ref: 12706898 Gene, 2003
